# Evaluation of the Impact of Virtual Reality-Enhanced Cardiac Rehabilitation on Depressive and Anxiety Symptoms in Patients with Coronary Artery Disease: A Randomised Controlled Trial

**DOI:** 10.3390/jcm10102148

**Published:** 2021-05-16

**Authors:** Sandra Jóźwik, Błażej Cieślik, Robert Gajda, Joanna Szczepańska-Gieracha

**Affiliations:** 1 Faculty of Physiotherapy, University School of Physical Education in Wroclaw, 51-612 Wroclaw, Poland; sandra.jozwik@awf.wroc.pl (S.J.); joanna.szczepanska@awf.wroc.pl (J.S.-G.);; 2 Faculty of Health Sciences, Jan Dlugosz University in Czestochowa, 42-200 Czestochowa, Poland; 3 Center for Sports Cardiology at the Gajda-Med Medical Center in Pultusk, 06-102 Pultusk, Poland; gajda@gajdamed.pl

**Keywords:** cardiovascular disease, CVD, depression, anxiety, cardiac rehabilitation, virtual reality

## Abstract

The aim of this study was to assess the efficacy of virtual reality (VR)-enhanced cardiac rehabilitation (CR) in reducing the intensity of depression and anxiety symptoms in patients undergoing phase II of CR in ambulatory conditions. One hundred participants (mean age 65.7 years) were divided randomly into two groups. Both groups took part in eight sessions of standard CR (three times per week). The experimental group was additionally supported by eight sessions of VR therapy using the VR TierOne device and the control group by eight sessions of Schultz Autogenic Training. The Hospital Anxiety and Depression Scale (HADS) was used as the primary outcome measure. The Perception of Stress Questionnaire was used as the secondary outcome measure. The data from 77 participants were subject to analysis. Post-intervention, in the experimental group, the overall HADS score was statistically significantly reduced by 13.5%, HADS-Depression by 20.8%, and the general stress level by 12.8% (*p* < 0.05). In the control group, the scores of the HADS, HADS-Anxiety and the general stress level were statistically significantly higher, by 4.8%, 6.5%, and 4.9%, respectively. VR-enhanced CR for individuals with cardiovascular disease reduced the level of anxiety and depression symptoms compared to standard CR.

## 1. Introduction

Cardiovascular disease (CVD) is the main cause of death in the world [1], whereas the most common mental disorder in health care is depression [2]. The presence of depression is associated with increased mortality, reduced quality of life, increased costs of health services, and reduced chances of returning to work [3]. According to the American Heart Association, depression is a negative prognostic factor at all stages of treatment of CVD [4,5].

Depression occurs more often in CVD patients (in as many as 20–45% of patients), and up to three times more often in patients with acute myocardial infarction (MI) than in the general population [2]. Apparently, this relationship also operates in a converse sense, as Halaris et al. noticed that people with depression have twice the risk of developing CVD [6]. They described the relationship between depression and CVD as resulting from over-stimulation of the sympathetic part of the autonomic nervous system leading to an inhibition of the vagus nerve and an increased heart rate. The authors also wrote about a noticeable dysfunction of the vascular endothelial cells in depression, which is a marker characteristic of both depression and CVD [6].

The presence of depression increases patient mortality by 77% over a period of 10 years after a percutaneous coronary intervention (PCI) [7]. In individuals with ischaemic heart disease (IHD) and depression, the risk of cardiac events is more than twofold higher compared to that of patients with IHD but with no mood disorders [8]. In patients with heart failure, depression is a significant and independent predictor of mortality [9]. In all the above diseases, psychosocial stressors can be both a cause and a consequence of cardiovascular events, at the same time being much more difficult to recognise than traditional risk factors for heart diseases [10].

In recent years, the treatment of symptoms of anxiety and depression has been increasingly aided by modern technologies, including those conducted in a virtual environment [11,12,13,14,15]. In cardiology, virtual reality (VR) increases accessibility by enabling consultations with other outstanding specialists in the field during procedures or by facilitating the planning of cardiac surgeries [16]. However, studies into the use of VR to improve the effects of rehabilitation have also become increasingly popular [17].

A literature review on the use of VR and video games in CR concluded that these new tools are a good complement to standard procedures, as they increase patient motivation and involvement in the rehabilitation process [18]. Research indicates that patients are happy to use such varied forms of training and report a desire to continue the exercise programme after their rehabilitation is complete [19,20]. 

One of the studies conducted aimed to assess the impact of a virtual walk on coronary artery bypass grafting (CABG) patients. The findings obtained confirmed that the use of VR accelerated the improvement of cardiovascular function [21]. In turn, pilot studies carried out by our team showed that VR therapy reduced significantly the severity of depressive symptoms and anxiety, as well as stress levels, in coronary artery disease (CAD) patients undergoing CR [22].

Therefore, the aim of this study was to assess the efficacy of VR-enhanced CR in reducing the intensity of depression and anxiety symptoms in CAD patients undergoing phase II CR in ambulatory conditions.

## 2. Materials and Methods

### 2.1. Study Design

The study design was set as a randomised controlled trial with a blinded outcome assessor. The study was conducted according to the guidelines of the Declaration of Helsinki. Participants were recruited from the PRO CORDE Cardiology Clinic in Poland. Participation in the study was fully voluntary, the patients provided a written consent to participate, and they were informed beforehand that they could quit the programme at any given moment without any consequences. The study protocol was approved by the Institutional Review Board of the authors’ affiliated institutions (no. 31/2019) and was registered a priori at ClinicalTrials.gov (NCT04313777) in March 2020.

### 2.2. Participants

One hundred participants undergoing phase II CR were recruited in the study. Fifty participants were allocated randomly to receive VR-enhanced rehabilitation (experimental group) and 50 to receive traditional CR (control group). Inclusion criteria: age 40–85 years; CAD; and undergoing phase II CR in ambulatory conditions. Exclusion criteria: cognitive impairment preventing self-completion of the research questionnaires; the presence of the following issues at the time of the examination or in the medical records: disturbances of consciousness, psychotic symptoms, bipolar disorder or other serious psychiatric disorders; initiation of psychiatric treatment or individual psychological therapy during the research project; contraindications for virtual therapy (epilepsy, vertigo, eyesight impairment); or patient’s refusal at any stage of the research project.

### 2.3. Intervention

Both groups took part in eight sessions of standard CR (three times per week). The experimental group was supported additionally by eight sessions of VR therapy using the VR TierOne device and the control group by eight sessions of Schultz Autogenic Training (SAT).

The standard CR consisted of two 40-min parts: interval training on a cycle ergometer and general fitness exercises, with a 15-min break in between. The intensity of the cycle ergometer interval training was prescribed individually based on the calculated heart rate reserve. The exercise heart rate is the resting heart rate plus 40–85% of the reserve. Interval training, while being as safe as continuous training, is superior in improving cardiorespiratory fitness in CVD patients [23]. The second part, general fitness exercises, consisted of exercises with the use of fitness equipment such as a treadmill, a pec fly machine, an elliptical trainer, a rowing machine, and a stepper. During the training, heart functions were monitored by electrocardiography (ECG). Additionally, three other separate measures of heart rate and blood pressure (before, during peak effort, and at the end of training) were taken. The final part of the training was a calming phase (about 10 min), followed, in the control group, by SAT played from a CD recording (three times a week for 20 min).

The experimental group, instead of the SAT, took part in a VR therapy session using the VR TierOne device (Stolgraf®, Stanowice, Poland). The VR set comprises a computer dedicated to processing 3D graphics, VR goggles (HTC VIVE PRO, 2017, New Taipei City, Taiwan) enabling the display of high-resolution images with high picture quality (90 Hz) and manipulators that transfer the patient’s hand movements into the VR world. The computer provides sufficient computing power for real-time transfer of the user’s movements into the virtual environment. The entire experience is made whole by surround-sound effects through the built-in headphones. Therapy designed to be used with this solution is based on the metaphor of a Virtual Therapeutic Garden where the patient is able to calm down and relax.

An important element of each session is the colouring of therapeutic mandalas (a new mandala every session). The patient’s engagement and efforts put into this task are rewarded as the garden, initially neglected and grey, regains its colours, energy, and beauty. VR TierOne engages all the patient’s senses (sight, hearing, kinaesthesia), deepening the process of immersion in the virtual world. The goals of the therapy are: calming and putting the patient in a state of psychophysical relaxation, recalling associations related to previous pleasant sensations, improving mood, reducing the level of anxiety, increasing the motivation to participate actively in the rehabilitation process, cognitive activation, and stimulation of the patient’s creativity.

The content of the VR therapy was developed by Joanna Szczepańska-Gieracha, a certified European Association of Psychotherapy therapist who specialises in the treatment of people with health problems. The project was supervised by Krzysztof Klajs, who is a professional supervisor and chairman of the Scientific Section of Psychotherapy of the Polish Psychiatric Association. The software was developed with a grant from the Polish National Centre for Research and Development (POIR.01-02.00-00-0134/16) and is described in an article about VR therapy in pulmonary rehabilitation [24] and late-life depression [25].

### 2.4. Outcome Measures

Initial assessment was carried out in both groups at the start of CR (week 0) and at a final assessment three weeks into rehabilitation (week 3). The outcome assessment was made by a blinded outcome assessor.

The Hospital Anxiety and Depression Scale (HADS) was used as the primary outcome measure. HADS is a tool to assess the level of depression and anxiety disorders. It consists of a 14-item scale scoring from 0 to 3 for each item. Seven items relate to anxiety (HADS-A), while the remaining seven relate to depression (HADS-D). The global scoring ranges from 0 to 42, with a cut-off point of 8/21 for anxiety and 8/21 for depression. The higher the score, the greater the anxiety or depression symptoms [26]. Cronbach’s *α* ranges from 0.78 to 0.93 for the HADS-A and from 0.82 to 0.90 for the HADS-D; test-retest correlation is *r* = 0.80 [27]. In our study, Cronbach’s *α* was 0.79 and 0.78 for the HADS-A, 0.77 and 0.83 for the HADS-D, 0.87 and 0.85 for the total HADS score, for pre- and post-intervention assessment, respectively.

The Perception of Stress Questionnaire (PSQ) was used as the secondary outcome measure. This tool serves to determine the general level of stress, as well as Emotional Tension, External Stress, and Intrapsychic Stress levels. The questionnaire comprises 27 statements, with 21 referring to individual components of stress and six to the lie scale. The respondent determines the degree to which a given statement concerns him or her, using a five-point Likert scale. The higher the score, the more severe the symptoms of stress. Cronbach’s *α* for the individual scale ranges from 0.69 to 0.80 [28]. In our study, Cronbach’s *α* ranged from 0.76 to 0.89.

### 2.5. Data Analysis

All analyses were performed using the STATISTICA 12 software (StatSoft, Palo Alto, CA, USA). Demographic characteristics are reported as mean with standard deviation (*SD*) for continuous variables and as percentages for categorical variables. Chi-square tests were used to assess significant associations between categorical variables. Normality of outcome measures was assessed with the Shapiro–Wilk test. Differences between the two groups were assessed using independent *t*-tests. To determine the effectiveness of the intervention, a two-way repeated measures Analysis of Variance (ANOVA) was applied. The correlation of improvement of mental state and selected variables was assessed with Pearson correlation coefficient. The level of significance was set at *α* < 0.05.

## 3. Results

### 3.1. Participants and Characteristics

**Figure 1:**
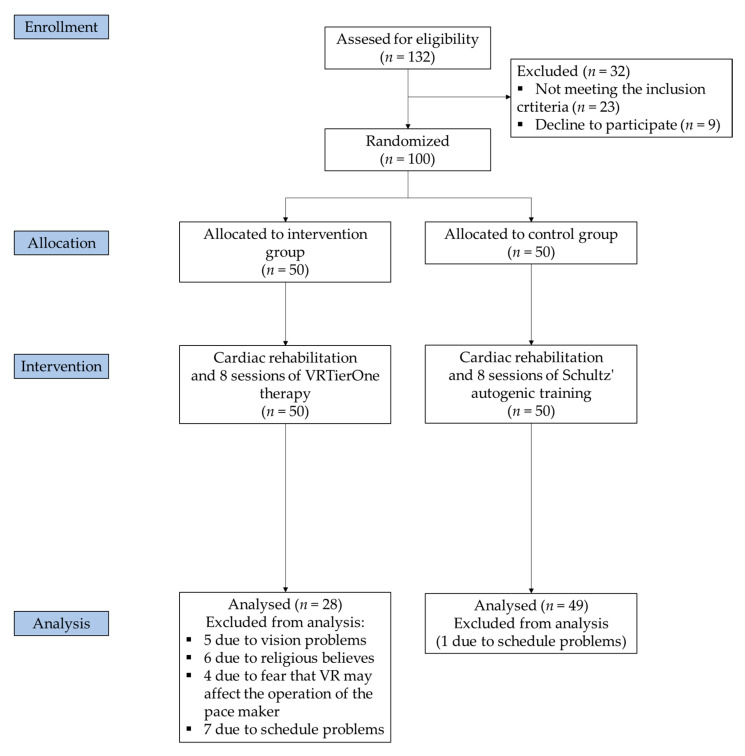
Study flow diagram.

Out of the 100 participants included, 23 dropped out of the study. Twenty-two dropped out of the experimental group: six due to religious beliefs, four due to fear that VR may affect the operation of the pacemaker, five due to vision problems, and seven due to schedule difficulties. From the control group, one participant dropped out due to schedule difficulties. For the final analyses, data were therefore available from 77 patients. Figure 1 presents the flow diagram of participants.

All patients had IHD. Either PCI or CABG procedures had been performed in 48.1% of the participants, 38.0% were diagnosed with MI, and 6.5% had an implanted device (e.g., a cardioverter-defibrillator, pacemaker, or heart stimulator). Table 1 illustrates participants’ baseline characteristics.

### 3.2. Assessment of Outcomes

Considering mental state examination, in the VR-enhanced rehabilitation group, except for HADS-A (*F*(1.27) = 1.46, *p* = 0.24, ηp^2^ = 0.05), all the examined parameters decreased significantly (Table 2). Total HADS score decreased significantly by 13.5% (*F*(1.27) = 5.60, *p* = 0.02, ηp^2^ = 0.17). HADS-D decreased significantly by 20.8% (*F*(1.27) = 10.10, *p* = 0.003, ηp^2^ = 0.27). General stress level assessed by the PSQ also decreased by 12.8% (*F*(1.27) = 14.88, *p* < 0.001, ηp^2^ = 0.35), Emotional Tension by 14.4% (*F*(1.27) = 12.88, *p* = 0.001, ηp^2^ = 0.32), External Stress by 12.8% (*F*(1.27) = 5.62, *p* = 0.02, ηp^2^ = 0.17) and Intrapsychic Stress by 11.0% (*F*(1.27) = 7.83, *p* = 0.009, ηp^2^ = 0.22).

In contrast, in the standard rehabilitation group, the assessed mental state parameters increased: total HADS by 4.8% (*F*(1.48) = 7.23, *p* = 0.01, ηp^2^ = 0.13), HADS-A by 6.5% (*F*(1.48) = 11.72, *p* = 0.001, ηp^2^ = 0.20), general stress level assessed by the PSQ by 12.8% (*F*(1.48) = 26.62, *p* < 0.001, ηp^2^ = 0.36), Emotional Tension by 6.9% (*F*(1.48) = 19.36, *p* < 0.001, ηp^2^ = 0.29), External Stress by 3.2% (*F*(1.48) = 8.00, *p* = 0.006, ηp^2^ = 0.14), and Intrapsychic Stress by 4.2% (*F*(1.48) = 11.64, *p* = 0.001, ηp^2^ = 0.19). All the results obtained differed significantly between the groups (Table 3).

Analysing correlation of improvement of mental state, it was shown that initial intensity of anxiety was correlated with change in HADS-A (0.48, *p* < 0.05), HADS-D (0.46, *p* < 0.05), and HADS (0.49, *p* < 0.05), initial intensity of depression with change in HADS-D (0.41, *p* < 0.05), initial total HADS score with change in HADS-A (0.40, *p* < 0.05), HADS-D (0.49, *p* < 0.05), and HADS (0.47, *p* < 0.05), and initial general stress level with change in PSQ (0.47, *p* < 0.05; Table 4). This means that the worse the patient’s mental state before CR was, the greater the improvement in the studied parameters. This relationship mainly concerned the VR group. No relationship was detected between the efficacy of standard CR or VR-enhanced CR and patient’s age or other sociodemographic factors.

## 4. Discussion

One of the most important objectives of comprehensive cardiac rehabilitation (CR) is to improve patients’ physical fitness together with their mental well-being [29]. Szczepańska-Gieracha et al. demonstrated that a clinically successful coronary artery bypass grafting (CABG) procedure and a properly conducted CR do not reduce the level of depression and anxiety disorders in individuals whose scores before rehabilitation exceeded the accepted standards (score of >10 points in the cognitive–affective subscale of BDI) [30]. The author emphasises that psychiatric symptomatology should be diagnosed as early as possible and that special treatment should be applied, as CR alone is not an effective method to treat mental problems.

Kustrzycki et al. arrived at similar conclusions in an eight-year-long observation conducted following a CABG procedure [31]. They found that a successful CABG procedure did not reduce depression and anxiety symptoms in either the short term (three months after surgery) or the long term (one year and eight years after surgery) [10]. Tulloch et al. suggested the need for routine checks on cardiac patients for depression and anxiety, as many such people are neither tested nor treated for their mental problems [32]. Unfortunately, such procedures are met with reluctance by staff and patients alike as being overly time-consuming and not very attractive [32].

Szczepańska-Gieracha, along with many other authors, sees the need for psychotherapeutic interventions in cardiac patients with increased symptoms of depression and anxiety [30,31,33,34]. Clinical practice shows that in such cases Schultz Autogenic Training, which is a standard method of psychological support in CR units, is not sufficient effectively to improve the mental state of cardiac patients [30,31]. In most facilities, SAT is played from a CD, which further reduces its attractiveness to patients, often causing them to give up participation in relaxation classes.

For several years now, we have seen a continuous increase in the implementation of new technologies, including VR in rehabilitation [35]. Results obtained showed that CR supplemented by VR therapy is more effective at reducing symptoms of depression and anxiety than the traditional CR approach offered in outpatient care to patients with heart diseases. This observation coincides with the observations of other authors who analysed the role of VR in the treatment of various mental disorders. In 2019, an attempt was made to develop VR-based anti-depression therapeutic techniques. Lindner et al. translated behavioural techniques used to treat depression into a VR experience comprising psychoeducation, behavioural activation, and social skills training. The authors concluded that VR is a clinically appropriate method for existing cognitive-behavioural techniques (CBT), and the VR-unique experiences may be employed to treat depression. Furthermore, they also stated that VR has a great potential to reduce the treatment gap for depression and can affect the mental health of society [36].

The results of our experiment support this thesis and are an important complement to the existing knowledge on the subject. The idea of the Virtual Therapeutic Garden is based on the assumptions of Erickson’s psychotherapy, and the most important metaphorical message lies in the symbolic care of a neglected garden that regains its life and beauty thanks to the involvement of the patient. This idea refers to the role of CR, which helps to restore a patient’s physical fitness and improve the work of the cardiovascular system despite the damage caused by cardiac disease. Our study showed that the metaphor employed was particularly effective in reducing the symptoms of depression (an improvement by 20%). This result confirms the findings of a pilot study in which, after the intervention, we achieved a 23% decrease in the intensity of depression symptoms [22]. However, it is worth noting that in the pilot study participants had a higher HADS-D score prior to starting therapy (9.00 in the pilot study vs. 6.14 in this study).

In contrast to our findings is a study by Vieira et al. (2018), where the authors demonstrated no change in the intensity of symptoms of depression after a VR intervention [20]. However, their study involved patients in phase III of CR and used a rehabilitation game based on an Xbox Kinect (Microsoft) device. The VR system used in the cited study did not allow such deep immersion as that provided by a head-mounted display (HMD). Also, more importantly, the rehabilitation game used by the authors was based on performing physical exercises with reinforced feedback with no psychotherapeutic elements. Another study that also used the Kinect system demonstrated that adding VR to conventional rehabilitation may reduce slightly the severity of depression symptoms during phase II of CR [37]. Perhaps extending functional VR rehabilitation by adding psychotherapeutic elements would allow for positive changes in both the functional state of patients and their mental condition.

The literature contains many data on the use of VR in treating anxiety, but without association with heart disease or CR. In this study, the anxiety level was the only parameter that did not change after intervention in the experimental group. However, it is worth noting that, at the same time, this parameter increased significantly in the control group. Therefore, we can assume that it was thanks to the intervention that it remained unchanged in the experimental group. Our pilot study achieved a significant reduction in this parameter [22]. However, similar to depression levels, the baseline anxiety level was significantly higher in the present study than in the pilot study. Perhaps there is a level to which the intensity of anxiety can be reduced through a VR intervention, and once it has been reached it is maintained at this level.

Interestingly, in this study, the decrease in the severity of perceived stress after the VR intervention was almost identical to that recorded in the pilot study [22]. In the current study, stress level decreased by 12.8% (55.93 vs. 48.75) and in the pilot study by 12.7% (64.73 vs. 56.47). Vieria et al. (2018), in their study using a less immersive Kinect system, did not demonstrate its effect on stress levels [20], whereas Gerber et al. (2017), who used an HMD VR system, stated that it had a relaxing effect, as shown by the vital markers of physical stress in patients staying in an intensive care unit [38]. The virtual therapy developed by our team places a great emphasis on breathing exercises and calming the patient during VR sessions, which resulted in a significant reduction in stress levels.

The analysis of the results obtained in the control group calls for separate consideration. It was found that in the group receiving standard CR the patients’ mental health did not improve despite regular physical exercises supervised by qualified medical staff. Total HADS score increased by 4.8%, with the most prominent increase, of 6.5%, recorded in anxiety levels. Also, a significant increase (of 4.9%) was recorded in stress levels, with the Emotional Tension parameter increasing by as much as 6.9%. The results described are very worrying and should prompt an intensive search for better solutions. It definitely seems that SAT is no longer an effective tool.

The last decade has seen such an acceleration in the pace of life and the number of various stimuli that never cease to affect us (such as our always-on mobile phones) that we have lost the natural ability to enter into the state of psychophysical relaxation that regenerates our mind and body. This may prove even more difficult for cardiac patients with severe anxiety and depression symptoms. Outpatient CR is “squeezed in” between work and household chores, and patients often quietly give up on relaxation classes to have more time for other things. Unfortunately, it is common practice in rehabilitation wards to play relaxation sessions or autogenic training from a CD, which patients often find unattractive and boring. In addition, cardiac patients, to a greater extent than others, find it difficult to stop the constant stream of thoughts to enable them to yield calmly to relaxation and may even feel increased irritation at the moments of “forced pause” in already unappealing relaxation classes.

The un-noticed constant haste, nervousness, and anxiety that are major causes of heart disease should be taken very seriously during each phase of CR. However, improving physical condition and cardiorespiratory fitness typically come to the fore, while dealing with psychological issues is still treated as a modest addition, or even marginal to therapeutic activities. In 2012, Szczepańska-Gieracha stressed that CR is not an effective treatment for depression and anxiety disorders in cardiac patients and called for the introduction of an early diagnosis and treatment of mental disorders in patients with heart disease [30]. Likewise, Tulloch et al. and Zeng et al. wrote about the need for screening tests to diagnose psychosomatic disorders and for the inclusion of new and pleasant treatments for mental disorders in addition to traditional therapies [32,39]. Goldstein et al. wrote that people with depression and anxiety disorders are more likely to develop acute MI and heart failure and are at increased risk of mortality [40]. The whole situation resembles a vicious circle. Although the presence of depression and anxiety disorders increases the risk of CVD, which also means that patients suffering from them need more help during treatment and rehabilitation, patients do not receive such help, which is confirmed by the results of our study seen in the control group and in data reported by other authors for many years.

Our study has several important limitations. Due to a simple pre-post design the study lacks a follow-up, which would capture long-term effects of our intervention. Also, a large number of dropouts in the experimental group is puzzling. Some patients raised concerns that such an advanced technology could interfere with pacemaker function or increase visual impairment in case of cataracts or glaucoma. This is an important issue to consider in further research on the use of VR in individuals with certain somatic diseases. Other participants were concerned that the Catholic church does not support psychotherapy, which is a concern specific to the Polish population and presumably would not be an issue in many other countries. Taking into account the above limitations, the results of our study are important but should be interpreted with caution.

## 5. Conclusions

Despite limitations, our study provided evidence that enhancing standard CR with therapy carried out in a virtual environment leads to a significant improvement in patients’ mental health. The intensity of visual, auditory, and kinaesthetic stimuli offered by VR therapy attracts patients’ attention, provides a welcome distraction from daily routine, and allows them effectively to slow down, calm down, and relax. The attractiveness of modern therapeutic methods confers a significant advantage over traditional techniques that require greater motivation and awareness on the part of the patient. The results of our study showed the potential benefits of VR therapy for people with heart disease, as well as its limitations, so further research in this area is very important.

## Figures and Tables

**Table 1:** Participants’ baseline characteristics.

Variable	Overall	VR-Enhanced Rehabilitation	Standard Rehabilitation	*p* Value *
**N**	77	28	49	-
Age, years (*SD*)	64.70 (8.03)	66.00 (9.73)	63.96 (6.89)	0.29
n (%) of females	42 (54.54)	17 (60.71)	25 (51.02)	0.41
Body mass, kg (*SD*)	77.68 (14.49)	75.50 (14.97)	78.91 (14.97)	0.32
Height, cm (*SD*)	165.75 (14.29)	164.54 (8.62)	166.45 (16.74)	0.58
BMI, kg/cm^2^ (*SD*)	29.25 (13.70)	27.79 (4.03)	27.65 (3.98)	0.49
IHD, n (%)	77 (100.00)	28 (100.00)	49 (100.00)	-
PCI/CABG	37 (48.05)	15 (53.57)	22 (44.90)	0.46
Diabetes, n (%) Body weight	22 (28.57)	8 (28.57)	14 (28.57)	0.99
Normal (BMI 18.5–24.9), n (%)	19 (24.67)	6 (21.43)	13 (26.53)	0.62
Overweight (BMI 25–29.9), n (%)	35 (45.45)	14 (50.00)	21 (42.86)	0.55
Obese (BMI > 30), n (%)	23 (29.87)	8 (28.57)	15 (30.61)	0.85
Education				
Elementary and vocational, n (%)	25 (32.47)	9 (32.14)	16 (32.65)	0.96
Secondary, n (%)	21 (27.27)	5 (17.88)	16 (32.65)	0.16
Higher education, n (%)	31 (40.30)	14 (50.00)	17 (34.69)	0.19
Marital status				
Married, n (%)	48 (62.34)	16 (57.14)	32 (65.30)	0.48
Single/divorced, n (%)	14 (18.18)	8 (28.57)	6 (12.25)	0.07
Widow, n (%)	15 (19.48)	4 (14.29)	11 (22.45)	0.38

BMI: body mass index; *SD*: standard deviation; IHD: ischaemic heart disease; PCI: percutaneous coronary intervention; CABG: coronary artery bypass grafting; * Chi-square test or *t* test, as appropriate.

**Table 2:** Primary and secondary outcomes.

Outcome	VR-Enhanced Rehabilitation	Standard Rehabilitation	*p* Value * (Between Groups)
HADS-total			
Before	13.71 (7.40)	13.57 (7.33)	
After	11.86 (6.58)	14.22 (7.11)	
Change	−1.86, −13.5% (−3.47 to −0.25)	0.65, 4.8% (0.16 to 1.14)	<0.001
*p* value	0.02	0.01	
HADS-A			
Before	7.57 (4.00)	7.22 (4.11)	
After	7.00 (3.56)	7.69 (4.02)	
Change	−0.57, −7.5% (−1.54 to −0.40)	0.47, 6.5% (0.19 to 0.75)	0.01
*p* value	0.47	0.001	
HADS-D			
Before	6.14 (3.77)	6.35 (3.91)	
After	4.86 (3.48)	6.53 (3.86)	
Change	−1.29, −20.8% (−2.12 to −0.46)	0.18, 2.8% (−0.16 to 0.52)	<0.001
*p* value	0.003	0.29	
PSQ			
Before	55.93 (19.32)	59.69 (17.55)	
After	48.75 (17.47)	62.63 (16.51)	
Change	−7.18, −12.8% (−10.99 to −3.36)	2.94, 4.9% (1.79 to 4.08)	<0.001
*p* value	<0.001	<0.001	
Emotional Tension			
Before	21.86 (7.28)	22.81 (7.11)	
After	18.71 (7.20)	24.39 (6.89)	
Change	−3.14, −14.4% (−4.94 to −1.35)	1.57, 6.9% (0.85 to 2.29)	<0.001
*p* value	0.001	<0.001	
External Stress			
Before	15.93 (6.06)	17.80 (5.93)	
After	13.89 (5.52)	18.37 (5.49)	
Change	−2.04, −12.8% (−3.80 to −0.27)	0.57, 3.2% (0.17 to 0.98)	<0.001
*p* value	0.02	0.006	
Intrapsychic Stress			
Before	18.14 (7.91)	19.08 (6.56)	
After	16.14 (7.05)	19.88 (6.30)	
Change	−2.00, −11.0% (−3.47 to −0.53)	0.79, 4.2% (0.33 to 1.26)	<0.001
*p* value	0.009	0.001	

Before and after variables are expressed as means (*SD*); change and differences variables as mean, % with 95% confidence interval (*CI*); * *t* test; HADS: Hospital Depression (D) and Anxiety (A) Scale; PSQ: Perception of Stress Questionnaire.

**Table 3:** Repeated ANOVA results.

	Variable	MS	*F* Value	*p* Value	ηp^2^
Time	HADS	12.96	3.22	0.08	0.04
	HADS-A	0.09	0.07	0.80	0.00
	HADS-D	10.86	8.49	<0.001	0.10
	PSQ	160.15	7.11	0.01	0.09
	Emotional Tension	22.00	3.75	0.06	0.05
	External Stress	19.10	4.39	0.04	0.06
	Intrapsychic stress	12.92	3.77	0.06	0.05
Time*Group	HADS	56.05	13.94	<0.001	0.16
	HADS-D	9.65	6.79	0.01	0.08
	HADS-A	19.18	14.99	<0.001	0.17
	PSQ	911.94	40.46	<0.001	0.35
	Emotional Tension	198.00	33.77	<0.001	0.31
	External Stress	60.56	13.91	<0.001	0.16
	Intrapsychic stress	69.64	20.33	<0.001	0.21

MS: mean square; HADS: Hospital Depression (D) and Anxiety (A) Scale; PSQ: Perception of Stress Questionnaire.

**Table 4:** Correlation of improvement of mental state in VR-enhanced rehabilitation and standard rehabilitation.

Outcome	VR-Enhanced Rehabilitation	Standard Rehabilitation
Age		
HADS-A	0.13	0.09
HADS-D	0.14	0.18
HADS	0.01	0.17
Stress	0.07	−0.16
Initial intensity of anxiety		
HADS-A	0.48 *	0.31 *
HADS-D	0.46 *	−0.07
HADS	0.49 *	0.10
Stress	0.18	0.10
Initial intensity of depression		
HADS-A	0.22	0.18
HADS-D	0.41 *	0.04
HADS	0.33	0.14
Stress	0.14	0.32 *
Initial HADS score		
HADS-A	0.40 *	0.21
HADS-D	0.49 *	−0.02
HADS	0.47 *	0.13
Stress	0.18	0.23
Initial general stress		
HADS-A	0.32	0.20
HADS-D	0.25	0.02
HADS	0.30	0.15
Stress	0.43 *	0.37 *

HADS: Hospital Depression (D) and Anxiety (A) Scale; * significant correlations at the level of *p* value < 0.05.

## Data Availability

Data are available from the corresponding author on a reasonable request.
